# Developing the Bias Blind Spot: Increasing Skepticism towards Others

**DOI:** 10.1371/journal.pone.0141809

**Published:** 2015-11-02

**Authors:** Fadwa B. Elashi, Candice M. Mills

**Affiliations:** 1 Educational Studies, Arab Open University, Amman, Jordan; 2 School of Behavioral and Brain Sciences, The University of Texas at Dallas, Richardson, TX, United States of America; Centre Hospitalier Universitaire Vaudois, FRANCE

## Abstract

Two experiments with eighty-eight 7- to 10-year-olds examined the bias blind spot in children. Both younger and older children rated themselves as less likely than a specific other (Experiment 1) or an average child (Experiment 2) to commit various biases. These self-other differences were also more extreme for biased behaviors than for other behaviors. At times, older children demonstrated stronger self-other differences than younger children, which seemed primarily driven by older children’s judgments about bias in others. These findings suggest that, although the bias blind spot exists as soon as children recognize other-committed biases, what changes over development is how skeptical children are towards others.

## Introduction

People berate others for their biases while remaining blissfully ignorant of their own [1, 2]. This *bias blind spot*–believing oneself to be far less biased than others—can be problematic given that biased decisions can have negative consequences. For instance, leaders of internationally conflicting countries frequently struggle to reach resolution, in part because each country sets a blind eye to their own biases while highlighting the biases of the other [1]. Being blind to one’s own biases can also lead to poor decision making, like a voter making decisions based on physical attractiveness instead of the relevant issues [3] and a landlord making decisions based on race instead of merit [4], all while being convinced of their accuracy. Yet, although the bias blind spot is pervasive in adults, next to nothing is known about how it develops or whether it even exists in children. The current experiments examine these issues.

Prior research suggests that aspects of the bias blind spot are present quite early in childhood: young children are often notoriously egocentric, neglecting to take the perspective of others while focusing on their own [5], and they attribute stability of positive traits more often to themselves than to others [6]. Crucially, however, this research only suggests that children will tend to see themselves more positively than others for non-biased behaviors—it says nothing regarding whether children, like adults, will have a particularly strong blind spot when it comes to thinking about *biased* behaviors.

For the bias blind spot to likely exist in children, it is presumed that children need to recognize the possibility of committing bias in the first place. Otherwise, how can they claim that others, and not themselves, commit biases? Past research has demonstrated that around age 7, children understand that people’s decisions can be biased because of relationships (e.g., a friend chooses a friend to be winner of contest; [7,8]), self-enhancement (e.g., a person claims to be smart to appear positive; [9]), self-interest (e.g., a person perceives him or herself to be the winner of a contest even though he or she was not the winner; [10]), or group membership (e.g., a teacher prefers students of the same race or gender group; [11]).

Although this research provides useful insight into understanding how children recognize bias, it has mostly been conducted by asking children about the biased decisions of *others*. However, given evidence of large self-other differences in the evaluation of biased behaviors in adulthood [1, 2, 13], a critical question remains: once children recognize that others can be biased, do they fall victim to the bias blind spot, and, if so, how does this change over development?

To gain a better understanding of the development of the bias blind spot (including if it even exists in children), two experiments were conducted examining children between the ages of 7 and 10. Children were presented with biases that previous research has shown they are able to recognize in others [8–12]. Mirroring research with adults [2] but using methods appropriate for children, participants in Experiments 1 and 2 heard explanations of biased and control (neutral, non-biased) behaviors and were asked to rate the likelihood that they and others would commit these behaviors. Control behaviors were included to determine whether children show an overall self-other difference (regardless of behavior type) or show stronger self-other differences to biased behaviors.

We expected that children would demonstrate the bias blind spot, rating others as more likely to commit biases than themselves. But our intuitions regarding what might change over development were unclear. One possibility was that there would be no age differences: thus, as early as children have been shown to recognize that bias exists in others, they might be less willing to admit bias existing in themselves. Another possibility, though, was that there would be age differences in how children thought about the self or others (or even for both). Specifically, in regards to thinking about the self, given research finding that younger children are often less sensitive than older children to social rules (e.g., racial categorization; [14]), it was possible that younger children would not recognize that people generally view committing biases as unacceptable. Thus, they might be more willing than older children to admit committing biases. In regards to thinking about others, it was possible that we would see increased skepticism of other people over development: past research has found that older children are more likely than younger children to generate explanations referring to the possibility that someone’s decision may have been skewed by bias [15]. Thus, older children may be more skeptical in general than younger children about the likelihood of others committing bias.

## Experiment 1

### Methods

#### Participants

Twenty 7- and 8-year-olds (*M*_*age*_ = 7.83 years, *SD* = .67; 11 females) and 20 9- and 10-year-olds (*M*_*age*_ = 9.75 years, *SD* = .48; 10 females) participated in this experiment. Participants were recruited from local elementary schools after providing written parental consent and received a small toy and certificate for their participation. Most participants were middle class and Caucasian.

#### Procedures

The protocol for this and the second experiment were approved by the internal subject IRB panel at The University of Texas at Dallas. The experimenter began the session by placing a cardboard folder between the child and herself, explaining that she could not see the child’s responses and that the child should “put the answers that are the truest for you”. So that children did not feel the need to answer in socially desirable ways, they were asked to circle their responses on an illustrated scale: a completely filled square represented “a lot”, a partially filled square represented “a little”, and an empty square represented “not at all”. The experimenter pointed to each image while reading the printed corresponding label. The images were used to help children pictorially understand the difference between each point on the scale.

Next, to help children familiarize with the experimental tasks and feel comfortable with using either side of the scale, children were read a positively-valenced (i.e., “Sometimes people like to play with their friends.”) and negatively-valenced story (i.e., “Sometimes people like to eat broccoli for every single meal, every single day.”). After hearing each story, the experimenter asked two questions. For the *self-rating questions*, children were asked “how much do you think you are like these people—a lot, a little, or not at all?” For the *other-rating questions*, children were presented with an expressionless, gender-matched character drawing and asked “how much do you think Paige (Paul) is like these people—a lot, a little, or not at all?” The order of the rating questions was counterbalanced. Responses were not examined for this portion of the experiment since the stories were solely designed to familiarize children with the experimental material.

Moving to the experimental tasks (adapted from [2]), children heard two types of stories: bias and control stories. The story characters were gender matched with the participant, and the stories were presented pseudorandomly. For the *bias stories*, children heard two stories for each bias that children of this age group have been previously shown to recognize in others (i.e., relationship, self-enhancement, self-interest, and group membership). To illustrate, for the relationship bias, children heard the following: “Sometimes people pick their best friend to be the winner of a contest even though someone else did the best because they want their friend to be happy.” For the *control stories*, children heard a total of four stories, depicting various non-biased behaviors that had been rated as neutral in terms of social acceptability. These stories were similar in nature to the ones presented in the beginning of the session to help children familiarize with the task. For example, children heard: “Sometimes people like to get gifts even when it’s not on their birthday because they like to get new things.” Again, the purpose of these stories was to determine if children show an overall self-other difference or more so to biased behaviors. Additionally, the control stories used in the current experiments were selected after children rated them as demonstrating the most neutral behaviors among a larger group of non-biased behaviors (c.f. [16]). Like the familiarization portion of the experiment, children were asked the self-rating questions and other-rating questions after each story.

### Results and Discussion

Participants' responses for each of the self- and other-rating questions were converted to a 3-point scale, where higher scores indicated greater similarity with the story characters and lower scores indicated weaker similarity with the story characters. Average scores were calculated for the self- and other-rating questions for each story type. No gender or order (of the rating questions) effects were found, and thus these variables have been excluded from the analyses described below.

A three-way mixed-design ANOVA with Target (self-rating questions, other-rating questions) and Story Type (bias, control) as the within subjects variable and Age (younger children, older children) as the between subjects variable was conducted. This revealed a main effect of target, *F*(1, 38) = 5.26, *p* = .03, η_p_^2^ = .12, and story type, *F*(1, 38) = 116.145, *p* < .001, η_p_^2^ = .85, as well as an interaction between these variables *F*(1, 38) = 14.09, *p* = .001, η_p_^2^ = .27.

**Fig 1:**
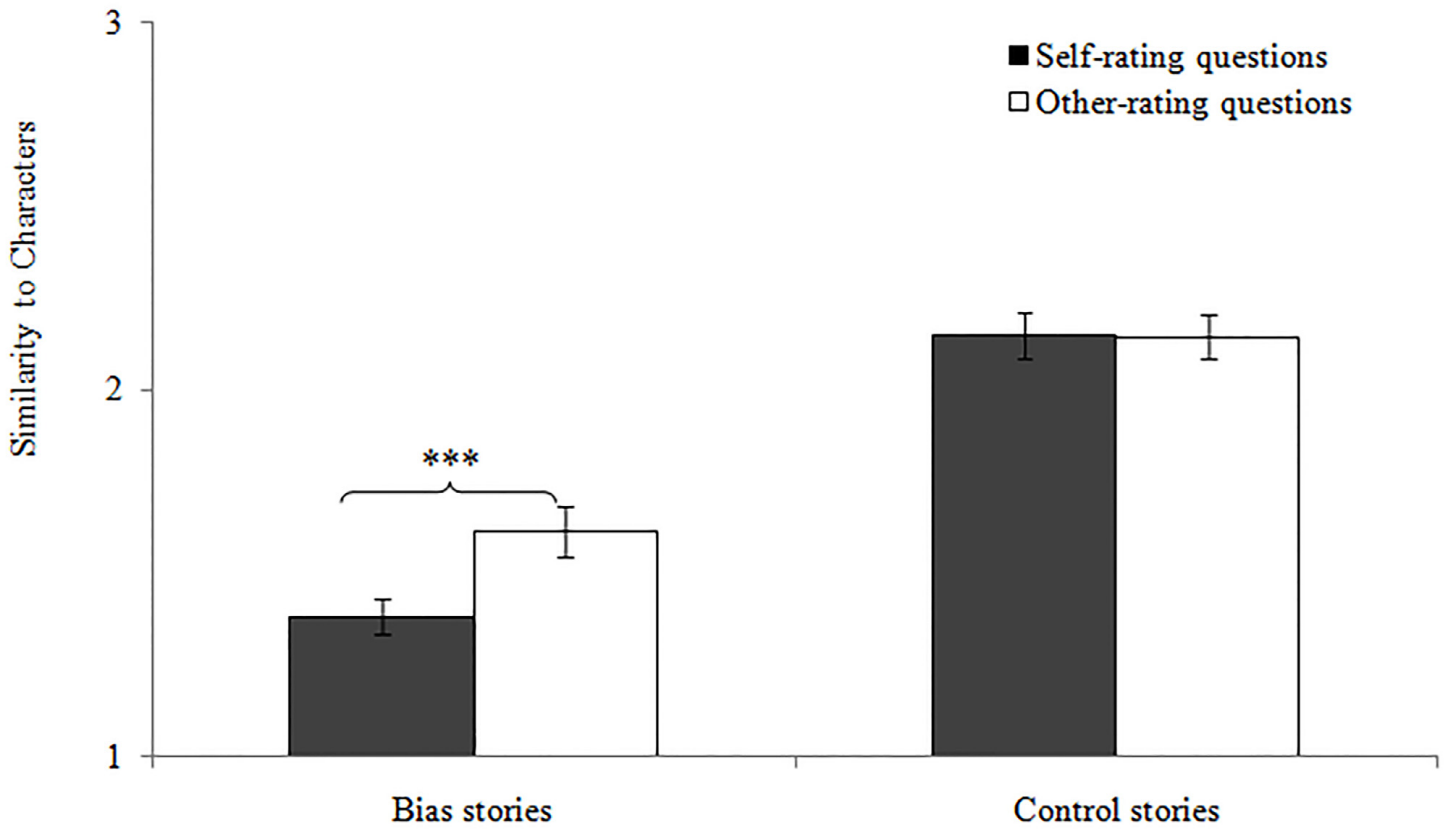
Means of the self- and other-rating scores for the bias and control stories in Experiment 1. The error bars represent the standard error. *** *p* < .001.

Follow-up analyses of the Target x Story Type interaction with Bonferroni corrections revealed that children did not demonstrate a significant self-other difference for the control stories, *t*(39) = .27, *p* = .789, even though they showed a significant self-other difference for the bias stories, *t*(39) = 4.52, *p* < .001 (see Fig 1). In fact, post-hoc analyses with a Bonferroni correction for multiple comparisons revealed that children demonstrated self-other differences across all bias types, *ts* > -3.97, *ps* < .025, except for a trending significance for the relationship bias, *t*(39) = -1.78, *p* = .083 (see Table 1 for all *t*-values).

**Table 1:** Means (standard deviations) for the self- and other-rating scores for each bias in Experiment 1.

	Self-rating score	Other-rating score	*t*
	Mean (SD)	Mean (SD)	
**Relationship**	1.51 (.46)	1.65 (.53)	1.77†
**Self-interest**	1.48 (.51)	1.70 (.65)	3.67**
**Self-enhancement**	1.24 (.47)	1.56 (.60)	3.97***
**Group membership**	1.30 (.50)	1.54 (.54)	2.35*

Scores ranged from 1 to 3, with higher scores indicating similarity with the characters demonstrating the bias and lower scores indicating dissimilarity with the characters demonstrating the bias. Stars indicate self- and other- rating score comparisons.

* p < .05,

** p < .01,

*** p < .001,

^†^ = .083.

**Fig 2:**
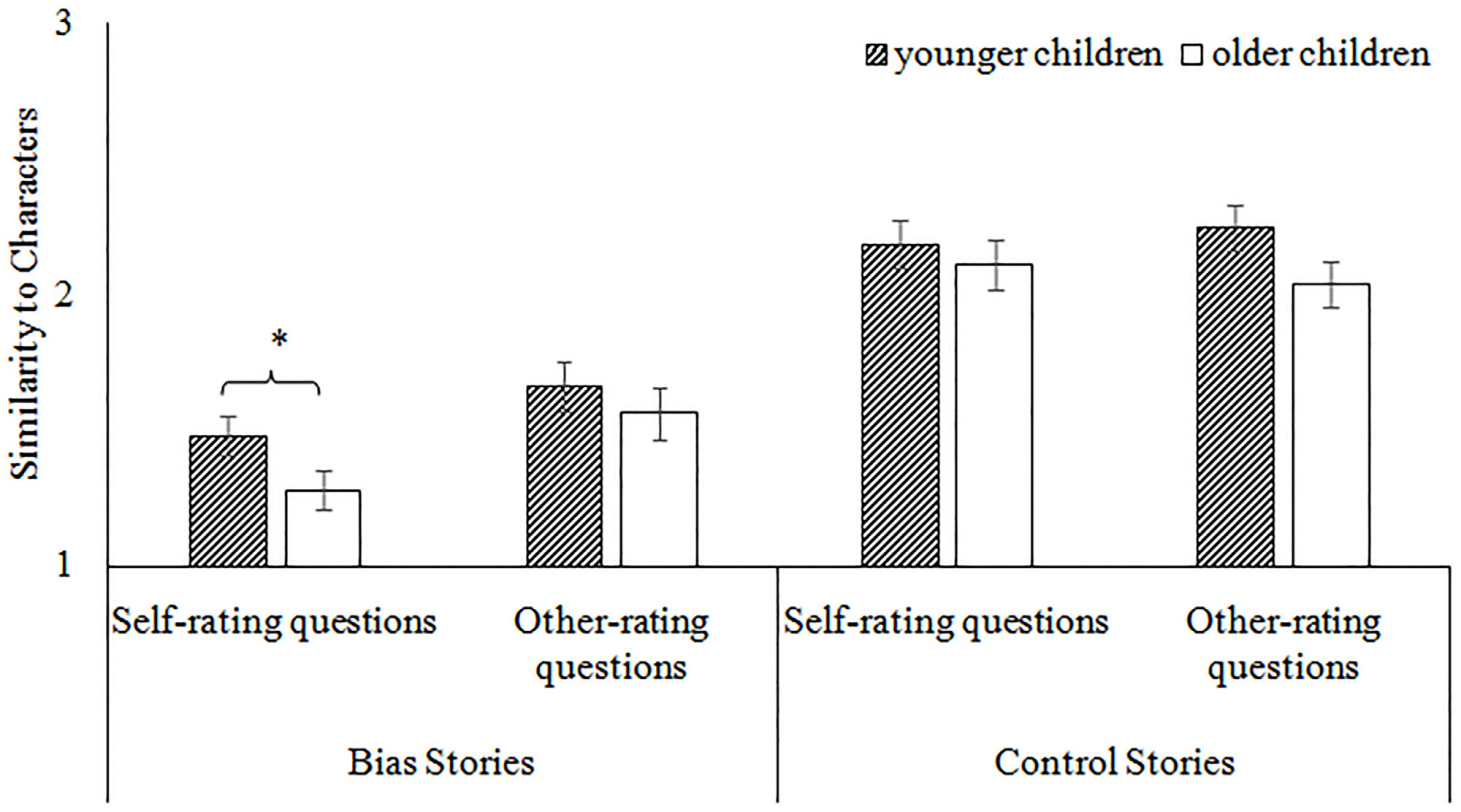
Means of the self- and other-ratings for younger and older children in Experiment 1. The error bars represent the standard error. *** *p <* .001, * *p <* .05.

Finally, a trend towards a significant interaction between all three variables was also found, *F*(1, 38) = 3.52, *p* = .068, η_p_^2^ = .09 (see Fig 2). For the control stories, there were no differences between the age groups’ self- and other-rating scores, *Fs* < 1.73, *ps* > .196. In contrast, for the bias stories, developmental differences were found for the self-ratings, *F*(1, 39) = 4.26, *p* = .046, such that younger children (*M* = 1.48, *SD* = .39) were more likely than older children (*M* = 1.28, *SD* = .19) to rate themselves as likely to commit biased behaviors. That said, no age differences were found for the other-ratings, *F*(1, 39) = .548, *p* = .464. Still, each age group demonstrated significant differences between the self- and other-ratings for the biased stories, *ts >* 2.32, *ps <* .04. Also, each group’s rating scores were significantly below the midpoint of the scale, *ts* > 3.06, *ps* < .006, supporting that children were not uncertain about their ratings. In sum, children of both age groups rated *others* as being more likely to commit biases than themselves. The crucial difference between the age groups is in how children rated themselves: younger children were still more willing than older children to admit that they were similar to the characters committing biases. These results are consistent with previous research suggesting that younger children may not be attuned to social convention as much as older children and thus more willing to admit to behaviors like biases [14].

That said, there are two issues to consider when interpreting these findings. First, participants evaluated a specific character (e.g., Paige or Paul) in this study. However, there is evidence to suggest that self-other differences are less prominent when participants evaluate a “specific” other than when they evaluate a more “average” other (e.g., the blind spot was weaker when college students were asked to rate how likely they think a classmate would commit biases than when asked to indicate how likely they think an average American would commit biases; [2]). Thus, children may also be more charitable to specific individuals than to an “average” individual, and this may explain the lack of age group differences for the other-ratings in this experiment.

Second, the wording of the self-rating and other-rating questions (“How much do you think you are (Paige/Paul is) like these people?”) may have emphasized the characters instead of the behaviors. And, given that minimal information was provided regarding the story characters, children may have had difficulty responding to rating questions about similarity to those characters.

Experiment 2, then, addressed both issues described above by asking children about others less specifically (i.e., “regular kid”) and rewording the questions so that they were framed around the behavior.

## Experiment 2

### Methods

#### Participants

Twenty-six 7- and 8-year-olds (*M*_*age*_ = 7.84 years, *SD* = .47; 13 females) and 21 9 and 10-year-olds (*M*_*age*_ = 9.95 years, *SD* = .64; 10 females) participated in this experiment. Participants were recruited from local elementary schools after providing parental consent and received a small toy and certificate for their participation. Most participants were middle class and Caucasian.

#### Procedures

The procedures for this experiment were identical to those of Experiment 1 with two exceptions. First, children were no longer presented with an image of the other, and they were asked to rate the likelihood that “a regular kid” instead of a specific other (i.e., Paige or Paul) would commit the behaviors presented in the stories. This is consistent with previous research in which adults were asked to indicate how likely they think “an average American” committed a biased behavior [2]. Second, rather than asking participants “how much do you think you are like these people?”, participants were asked, “How much do you think you do (a regular kid does) this?” To ensure that children understood the intended definition of “regular kid” in the context of this question (i.e., average), all children were asked to indicate what they think a “regular kid” means. All children demonstrated a clear understanding of the term (e.g., “average”, “normal”, “other kid”). These responses are consistent with previous research examining children’s (4^th^, 6^th^, and 8^th^ graders) concept of averages, one of which included “reasonableness” [17].

### Results and Discussion

Participants' responses were scored and calculated similarly to Experiment 1. No gender or order (of the rating questions) effects were found, and thus these variables have been excluded from the analyses described below.

A three-way mixed-design ANOVA with Target (self-rating questions, other-rating questions) and Story Type (bias, control) as the within subjects variable and Age (younger children, older children) as the between subjects variable was conducted. This revealed a main effect of target, *F*(1, 45) = 69.5, *p* < .001, η_p_^2^ = .61, and story type, *F*(1, 45) = 40.4, *p* < .001, η_p_^2^ = .47, as well as an interaction between these variables, *F*(2, 90) = 57.8, *p* < .001, η_p_^2^ = .56.

**Fig 3:**
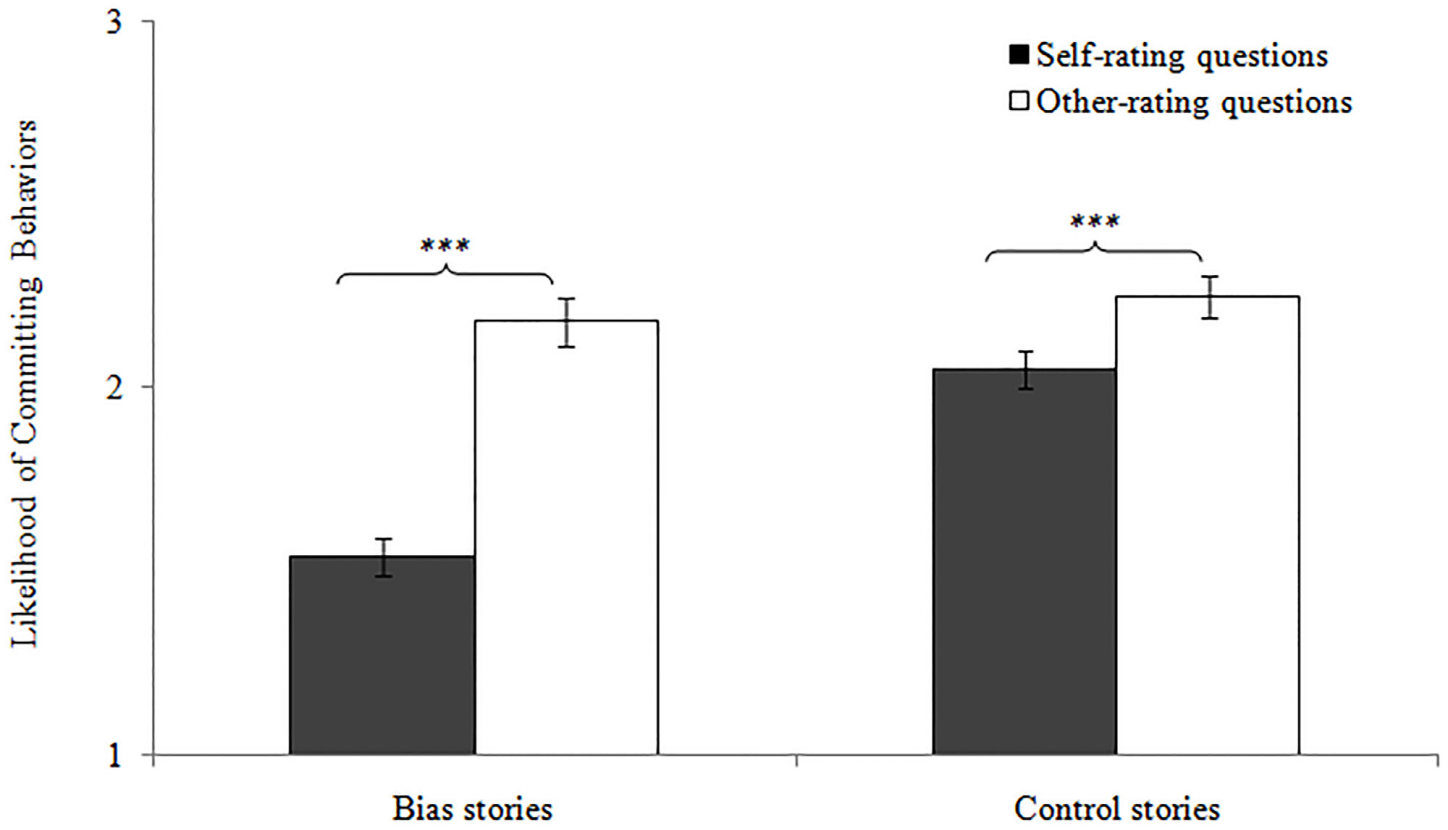
Means of the self- and other-rating scores for the bias and control stories in Experiment 2. The error bars represent the standard error. *** *p* < .001

Follow-up analyses of this interaction with Bonferroni corrections revealed that children demonstrated self-other differences for both the bias and control stories, *ts* > 3.74, *ps* < .001 (see Fig 3), but the differences were significantly *greater* for the bias stories (*M* = .63, *SD* = .45) than for the control stories (*M* = .20, *SD* = .36), *t*(46) = 6.83, *p* < .001. In fact, children demonstrated self-other differences across all bias types, *ts* > -8.93, *ps* < .0001 (see Table 2 for all *t*-values), and the self-other differences for the bias stories were stronger in Experiment 2 (*M* = .63, *SD* = .44) than in Experiment 1(*M* = .23, *SD* = .32), *t*(85) = 4.63, *p* < .001.

**Table 2:** Means (standard deviations) for the self- and other-rating scores for each bias in Experiment 2.

	Self-rating score	Other-rating score	*t*
	Mean (SD)	Mean (SD)	
**Relationship**	1.81 (.57)	2.24 (.61)	4.34***
**Self-interest**	1.54 (.62)	2.17 (.67)	7.03***
**Self-enhancement**	1.30 (.45)	2.06 (.63)	8.93***
**Group**	1.52 (.40)	2.19 (.57)	7.12***

Scores ranged from 1 to 3, with higher scores indicating likelihood of committing the bias. Stars indicate self- and other- rating score comparisons.

*** p < .001.

Additionally, although no differences were found in children’s rating of *others* per story type, *t*(46) = 1.54, *p* = .132, there were differences in how they evaluated the *self*, *t*(46) = 8.60, *p* < .001—children rated themselves as less likely to commit biased behaviors (*M* = 1.54, *SD* = .36) than control behaviors (*M* = 2.05, *SD* = .35). These findings suggest that, although self-other differences were observed for the control stories, they were more extreme for the bias stories.

**Fig 4:**
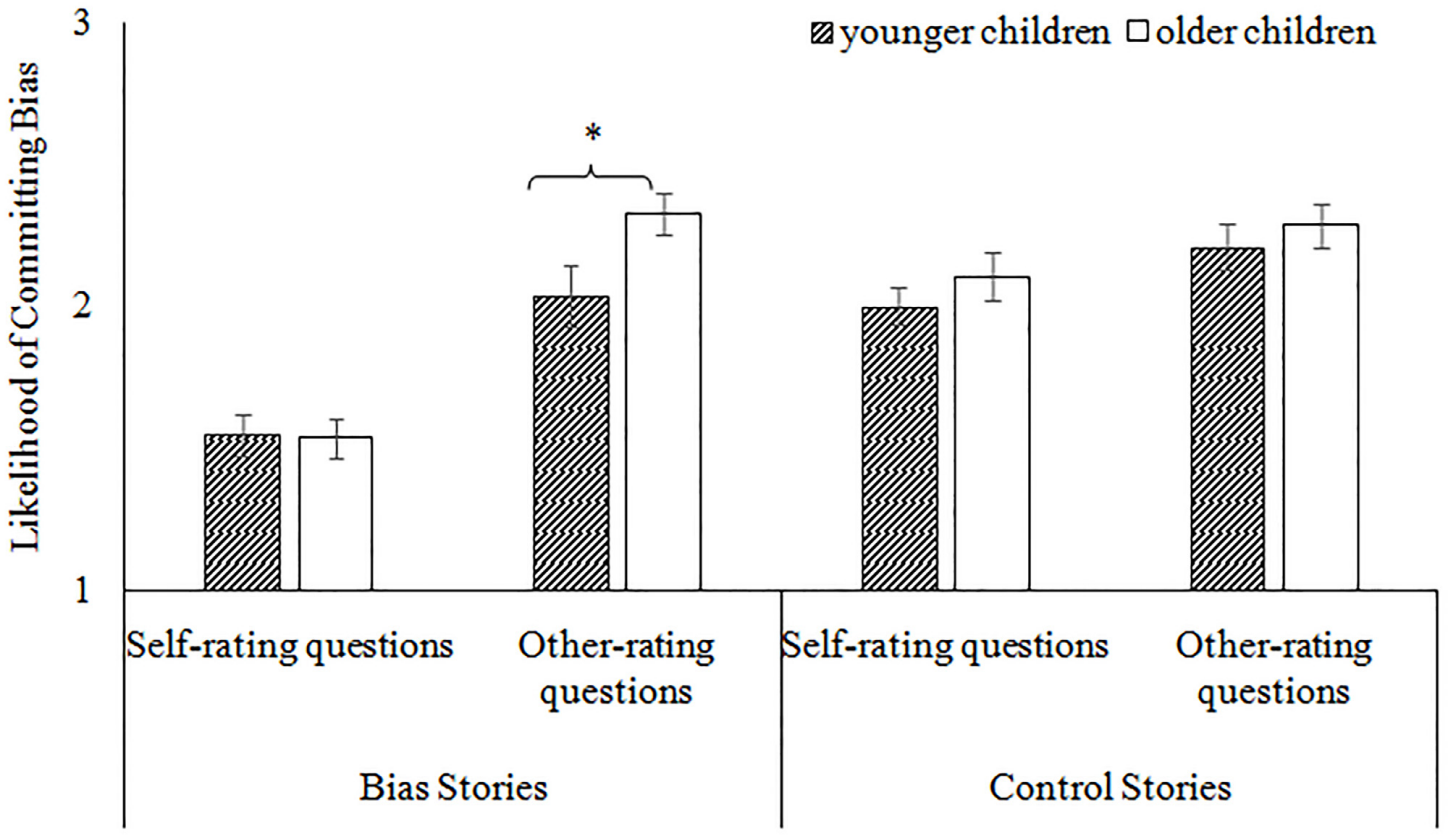
Means of the self- and other-ratings for younger and older children in Experiment 2. The error bars represent the standard error. * *p <* .05, *** *p* < .001.

Finally, an interaction between all three variables was found, *F*(1,45) = 8.1, *p* = .007, η_p_^2^ = .15. For the control stories, there were no differences between the age groups’ self- and other-rating scores, *ts* < 1.04, *ps* > .305. In contrast, for the bias stories, younger and older children only provided similar ratings when asked about the likelihood that *they* would commit a bias, *t*(45) = .117, *p* = .907, but provided different ratings when asked about the likelihood that *others* would commit a bias, *t*(45) = 2.14, *p* = .038 (see Fig 4). Specifically, older children (*M* = 2.33, *SD* = .34) were more likely than younger children (*M* = 2.04, *SD* = .54) to rate others as likely to commit biased behaviors. In fact, for the *self*-rating questions, both age groups’ ratings were significantly below the mid-point of the scale, *ts* > 5.94, *ps*< .001. In contrast, for the *other*-rating questions, older children’s ratings (*M* = 2.33, *SD* = .34) were significantly above the mid-point, *t*(20) = 4.40, *p* < .001, and younger children’s scores (*M* = 2.04, *SD* = .54) were no different than the mid-point, *t*(25) = .37, *p* = .717. We discuss these findings in further detail in the General Discussion, but briefly it suggests that children were not uncertain about their ratings of the self and other. Still, each age group demonstrated significant differences between the self- and other-ratings for the biased stories, *ts >* 6.16, *ps <* .001. These findings suggest that while both age groups demonstrated a bias blind spot, over development, children become more critical regarding the likelihood of the average other engaging in biased behaviors.

## General Discussion

The main aim of the current research was to examine the development of the bias blind spot. Findings from our experiments provide evidence to suggest that the phenomenon examined among adults begins as early as when children begin to recognize that bias is even possible [7–12]. Specifically, children as young as 7 years of age demonstrated a bias blind spot across both experiments, rating others as more likely than themselves to commit biased behaviors. Moreover, children seemed to demonstrate this blind spot specifically to biased behaviors—no or weak self-other differences were found when examining other neutral behaviors used here, mirroring past research findings with adults [2].

We believe it is likely that the same mechanism responsible for the bias blind spot in adults is also at least partially responsible for the blind spot in children. Specifically, people tend to believe that they can reflect on their thoughts and find evidence of how they have formed their beliefs. However, because biased behaviors often occur at a more unconscious level [18, 19], when people attempt introspection, they fail to find traces of bias (i.e., *introspection illusion*; [20–21]. Interestingly, children do not seem to reflect on the importance of introspection and underlying thought processes on decision-making until around age 7 [22], which seems to contribute to them recognizing that bias is even possible. In other words, as soon as children recognize that people’s conscious and unconscious thoughts can lead to bias, they, like adults, may place too much emphasis on introspection, finding themselves accusing others of bias while remaining convinced of their own objectivity.

Still, there is room for developmental change in the bias blind spot, and our research provides evidence into the nature of that change. First, we found that, in some circumstances, younger children were more willing than older children to report engaging in bias. It is possible that younger children are less hesitant to report committing biases because they, compared to older children, may be less attuned to the social rules of admitting to behaviors with negative associations [14]. Indeed, college students’ willingness to correct for a bias is related to how the bias may be perceived, with students more likely to seem aware of and correct for biases if doing so will make them appear in a positive light [23]. Thus, younger children’s self-ratings may have been influenced by their perceptions of the behaviors, leading to a greater willingness to admit to biased behaviors than their older counterparts.

It is also possible that younger children’s willingness to report committing bias may depend in part on how the question is asked. In Experiment 1, children were asked to rate how similar they were to the characters committing the behaviors, and younger children reported greater similarity to those characters than older children. In Experiment 2, children were asked to simply rate how likely they were to commit the behavior, and both younger and older children reported low levels of engaging in those behaviors. Asking children if they are similar to characters in a story rather than asking if they actually commit the behaviors may have led younger children to believe that it is acceptable to rate themselves as like the characters. That said, recall that both younger and older children’s rating scores in Experiment 1 were significantly below the mid-point of the scale. This seems to suggest that, although younger children were more willing than older children to admit to biases in Experiment 1, they still did not see themselves (and even others) similar to the characters of the stories (hence the low rating scores). Future research will need to further examine the factors that influence children’s willingness to admit to bias in themselves.

Second, we found that older children were less forgiving towards others than younger children. It is important to note that this developmental finding was only true when children were asked to rate a “regular kid” (Experiment 2) rather than a specific character (e.g, Paige or Paul; Experiment 1). This suggests that, although the bias blind spot exists as soon as children recognize bias may be plausible, it is possible that one thing that changes over development is how cynical children—and adults—are towards others in *general*. Indeed, unlike younger children, older children’s average rating score for others in Experiment 2 was above the mid-point of the scale, illustrating the strength of their bias blind spot by providing extreme ratings of others compared to their younger counterparts. One possibility for this increased cynicism towards others may be because older children have had more opportunities than younger children in which they had to doubt sources of misinformation.

We believe that this developmental finding surfaced only in Experiment 2 because of the change in our reference to the “other” from a specific sense (i.e., Paige or Paul) to a general sense (i.e., regular kid). Indeed, research with adults report a more prominent bias blind spot when participants are asked to indicate how likely they think an *average* American would commit biases than when asked about a specific classmate [2], and this was also true for our experiments. One explanation for this finding is that people may compare specific individuals (i.e., a classmate) to themselves (“a classmate is more like me than a complete stranger”) and thus rate them more favorably than other general individuals.

There are likely to be other factors that influence the degree to which children demonstrate self-other differences in attributions of biased behavior. For instance, it is likely that more general developmental changes influence children’s judgments about bias. Recent research has found that advances in social cognitive skills (e.g., interpretive theory of mind) correlate with the ability to recognize that a specific claim may be skewed by bias [24]. The ability to reason more deeply about the factors that can influence the thoughts and beliefs of others may also lead children to be more capable of recognizing how frequently other people’s decisions may be influenced by bias. Although these abilities may help children make attributions of bias in others, they are unlikely to help children recognize that they themselves have been the target of bias (see [25] for related research on perceiving oneself to be the target of discrimination), nor to recognize the possibility that they themselves are biased, given the power of the introspection illusion.

In sum, the current findings provide evidence that the bias blind spot has apparent roots in childhood. Indeed, children perceive others as more likely than themselves to commit biased behaviors as soon as they recognize that bias exists, and this judgment does not appear to be solely due to an overall perception that the self is better than others. That said, the nature of these self-other differences changes over development: while younger children sometimes admit to committing biases, older children are less willing to do so and are often more harsh than younger children on their evaluations of others'' likelihood of committing biases. Ultimately, these findings support that skepticism towards others looms large even in childhood and, thus, provide a foundation for future research aimed at improving conflict resolution and decision making.

## Supporting Information

S1 Dataset Dataset for Experiments 1 and 2. (XLSX)
